# High User Control in Game Design Elements Increases Compliance and In-game Performance in a Memory Training Game

**DOI:** 10.3389/fpsyg.2015.01774

**Published:** 2015-11-20

**Authors:** Aniket Nagle, Robert Riener, Peter Wolf

**Affiliations:** ^1^Sensory-Motor Systems Lab, Department of Health Sciences and Technology, ETH ZurichZurich, Switzerland; ^2^Spinal Cord Injury Center, Balgrist University HospitalZurich, Switzerland

**Keywords:** game design, motivation, in-game performance, frequency of game play, n-back, tablets, user-control, home-based training

## Abstract

Computer games are increasingly being used for training cognitive functions like working memory and attention among the growing population of older adults. While cognitive training games often include elements like difficulty adaptation, rewards, and visual themes to make the games more enjoyable and effective, the effect of different degrees of afforded user control in manipulating these elements has not been systematically studied. To address this issue, two distinct implementations of the three aforementioned game elements were tested among healthy older adults (*N* = 21, 69.9 ± 6.4 years old) playing a game-like version of the n-back task on a tablet at home for 3 weeks. Two modes were considered, differentiated by the afforded degree of user control of the three elements: user control of difficulty vs. automatic difficulty adaptation, difficulty-dependent rewards vs. automatic feedback messages, and user choice of visual theme vs. no choice. The two modes (“USER-CONTROL” and “AUTO”) were compared for frequency of play, duration of play, and in-game performance. Participants were free to play the game whenever and for however long they wished. Participants in USER-CONTROL exhibited significantly higher frequency of playing, total play duration, and in-game performance than participants in AUTO. The results of the present study demonstrate the efficacy of providing user control in the three game elements, while validating a home-based study design in which participants were not bound by any training regimen, and could play the game whenever they wished. The results have implications for designing cognitive training games that elicit higher compliance and better in-game performance, with an emphasis on home-based training.

## Introduction

### Games for cognitive training

In the past two decades, computer games have been increasingly used in improving the life of the elderly, who are a growing percentage of the general population (United-Nations, 2014), and account for increasing healthcare costs (Hurd et al., 2013; Wimo et al., 2013). Playing games in general is a positive activity associated with successful aging (Allaire et al., 2013). Additionally, playing games has been found to produce improvements in specific areas like working memory (Karbach and Verhaeghen, 2014; Ballesteros et al., 2015), executive functions (Kizony et al., 2012), attention (Smith et al., 2009), self-esteem (Danowski and Sacks, 1980; McGuire, 1984), etc. Working memory is an especially important part of cognition, since it is a key determinant of many higher-order cognitive functions (Brehmer et al., 2012) and is essential to maintaining a healthy and independent lifestyle (Borella et al., 2010).

A lot of research, therefore, has been devoted to designing cognitive training games targeted both at persons with mild cognitive impairments (MCI) and the healthy elderly (Kueider et al., 2012). The game industry has not lagged behind in commercializing cognitive training: several games, such as Brain Age (Nouchi et al., 2012), Cogmed (Chacko et al., 2014), Lumosity (Hardy et al., 2011) have emerged in the last decade. These commercial off-the-shelf games claim positive effects on cognitive functions (Nouchi et al., 2012; Chacko et al., 2014), although there is some amount of skepticism about these claims, with the methods argued not to be theoretically grounded (Gibson et al., 2012), or the results found not to be replicable (Shipstead et al., 2012; Smith et al., 2013).

### Different implementations of game design elements

Games for cognitive training, commercial or otherwise, are often augmented with specific game design elements to make the games more enjoyable and effective. Prominent examples include difficulty adaptation (Westerberg et al., 2007; Brehmer et al., 2012), storytelling and narrative elements (Padilla-Zea et al., 2014), competition (Burguillo, 2010), multiplayer features (Dede et al., 2005), rewards (Anguera et al., 2013), visual themes (Katz et al., 2014), etc. In general, games, for cognitive training or otherwise, consist of a particular configuration of game elements, and the way those elements are implemented can have a large impact on the enjoyment value (Yee, 2006a) and, in case of cognitive training games, potentially impact effectiveness of the game. While a few studies have looked at the effect of including individual elements in cognitive training games (Brehmer et al., 2012; Katz et al., 2014; Ninaus et al., 2015), there is a lack of research about the effect of different implementations of the elements.

An important overlooked aspect in cognitive training games is the provided degree of *user control*, i.e., the extent to which users can manipulate the training task and the game environment. Empowering users by providing them control over elements of a game can make the gaming experience more desirable and productive (Graesser et al., 2009). Allowing users to control individual game elements causes the *locus of control* to reside with the user, consequently leading to the training activity to become more user-centered and therefore engaging (Stapleton, 2004). The provided degree of user control can be used to demarcate implementations of three game elements, namely difficulty adaptation, rewards, and visual theme, into two broad types: implementations that provide a high degree of user control (“USER-CONTROL”) and those that provide a minimal degree of user control, with most game decisions being taken automatically by an algorithm (“AUTO”). These two modes form the *extrema* of user control, with USER-CONTROL providing very high control over elements, and AUTO providing very less. Delving into the three elements in detail makes it apparent that implementations at the two extrema can affect enjoyment and training effect of games in different ways.

#### Difficulty adaptation

Difficulty adaptation involves dynamically adjusting difficulty of the game during gameplay. It can be done automatically by the game based on some criteria, of which the most common is to match difficulty with user skill, since such matching is known to heighten motivation and boost performance (Csikszentmihalyi, 1990). Termed dynamic difficulty adjustment (DDA) (Hunicke, 2005), this technique has been used in numerous cognitive training games (Tárraga et al., 2006; Westerberg et al., 2007; Imbeault et al., 2011; Brehmer et al., 2012). Although DDA has been used extensively, being compared favorably against having no difficulty adaptation (Brehmer et al., 2012), it has its share of problems. For DDA to function correctly, user performance must be derived or predicted with some accuracy, which is not always possible (Wallner and Kriglstein, 2012; Loh et al., 2015). Additionally, the relationship between task difficulty and enjoyment is not strictly linear (Harter, 1978; Bostan and Öğüt, 2009), and therefore a constant linear increase in difficulty, brought on by a presumably constant linear increase in performance, might not be enjoyable, and, in fact, could be tiring (Qin et al., 2010; Alexander et al., 2013). An alternative to DDA is to give users the control of difficulty adaptation (Desurvire and Wiberg, 2009; Bedwell et al., 2012), which can lead to an enhanced sense of autonomy and consequently higher enjoyment (Ryan and Deci, 2000). While giving users the control of difficulty has been tried in games before (von Bastian and Eschen, 2015), it has been suggested that complete user control of difficulty would lead to sub-optimal training, since users tend to set a low difficulty level for themselves, and that a better strategy might be to combine user control with a recommendation from the game about an appropriate difficulty change (Nagle et al., 2014a). Such a technique of combining user control of difficulty adaptation with game advice has not been systematically tested before, and was therefore compared against DDA.

#### Rewards

In-game reward is another design element that can increase the enjoyment value of games (Wang and Sun, 2011). While some kinds of rewards, like score, are displayed continuously, others, like a feedback message, are displayed after finishing a round or level (Wang and Sun, 2011). Rewards in games are always given automatically based on user actions or user performance; the idea of allowing user control in rewards seems counter-intuitive, because the value of a reward derives mostly from the fact that it is given by an external entity (Deci et al., 1999). However, it can be argued that if the action that triggers rewards is under a high degree of user control, then users can indirectly control when they get rewards. In games, the quantum of reward is often directly linked to difficulty (Hunicke, 2005). Having a high degree of control of difficulty can then lead to users gaining control of when they are rewarded. Additionally, varying the frequency of rewards so that the next reward-giving instance becomes unpredictable, is known to be a factor of motivation (Howard-Jones and Demetriou, 2009). Using this principle, rewards can be *scheduled* to be given not at fixed intervals, but at unpredictable intervals. Known as the *variable-ratio* schedule, this method has been successfully applied in games (Siang and Rao, 2003; Yee, 2006b; King et al., 2010; Nagle et al., 2014b). Although the variable-ratio schedule has been used in games before, the effect of linking reward frequency to user-controlled difficulty has not been studied before, and was therefore compared against automatic display of feedback messages.

#### Visual themes

In cognitive training games for older adults, visual themes are presented either as part of the training task (De Schutter and Vanden Abeele, 2008; Gerling et al., 2012), or as part of background graphics to enhance the motivation value of the games (Smeddinck et al., 2013). Design recommendations for games targeted at older adults often consider visual themes only in the context of older adults' reduced visual and cognitive acuity (Ijsselsteijn et al., 2007; Gerling et al., 2010). However, visual themes, like difficulty, can be manipulated to increase enjoyment (McLaughlin et al., 2010; Smeddinck et al., 2013). One aspect of such manipulation which has not been studied in detail is the effect of allowing older adults to select a visual theme in a game. Giving users the choice of selecting a visual theme allows them to experience a sense of control over the gameplay, potentially enhancing their autonomy and increasing enjoyment (Ryan and Deci, 2000). Giving such a choice has been suggested to be a factor of motivation among older adults (Vasconcelos et al., 2012), and therefore can also influence the training effect of games. Thus, the effect of user control of visual theme was compared against the game automatically assigning a theme.

### Home-based training

With advances in mobile technologies, games are now widely available as applications playable on mobile phones and tablets (Vasconcelos et al., 2012; Werner et al., 2012; Oei and Patterson, 2013). A parallel development is the advent of home-based training (Shatil et al., 2010, 2014; Gigler et al., 2013), where the primary aim is to prevent the onset of cognitive impairments, rather than rehabilitation. A common feature in home-based training studies is the presence of a fixed training schedule, whereby participants were instructed to use the training software a certain number of times a week, which does not always result in an enjoyable training experience, or even in increased training effect (Green and Bavelier, 2008). On the other hand, giving persons the freedom to choose when and where they want to do a task enhances their autonomy and hence performance (Langfred and Moye, 2004). Hence, a home-based, regimen-free study design was used in the present work in which the game was played on a tablet.

### Aims

High enjoyment (Grahn et al., 2000; Blunsdon et al., 2003; Mitchell et al., 2005; Gomez et al., 2010) and long play duration (Ballesteros et al., 2015) are crucial elements in increasing the effectiveness of training games. However, the actual effectiveness of a cognitive training game, generally characterized by performance on the actual training task (Greitzer et al., 2007) can depend on factors other than enjoyment and play duration (Wechselberger, 2013), and also needs to be measured. To address these issues, a working memory training game based on the n-back task was designed and augmented with two modes: USER-CONTROL, which provided a high degree of user control of difficulty adaptation, rewards, and visual themes, and AUTO, which automatically adjusted or set the three elements. Two aspects were considered: compliance (measured by motivation, total duration of play, and frequency of play), and in-game performance on the trained task. The following research questions were postulated:

**Q1**. Are there significant differences in motivation, total duration of gameplay, and frequency of gameplay between USER-CONTROL and AUTO?

**Q2**. Are there significant differences in in-game performance on the trained task between USER-CONTROL and AUTO?

It must be emphasized that since the three game elements were tested together, the present study was not expected to determine the contribution of the individual elements. Thus, the primary aim was to investigate the overall effect of a specific joint set of game element implementations.

## Materials and methods

### Participants

Twenty-one participants were recruited through a presentation at an information lecture for seniors (average age = 69.9 years, SD = 6.4 years, 10 females). Inclusion criteria were autonomously living older adults, aged above 60, with a mini-mental state examination (MMSE) (Folstein et al., 1975) score greater than or equal to 27. Participation was voluntary and not reimbursed. Written informed consent was collected from the participants prior to the study and participants were informed that they could drop out of the study at any time. Prior to starting the study, ethics approval was obtained from the institutional ethics committee.

### Protocol

**Figure 1:**
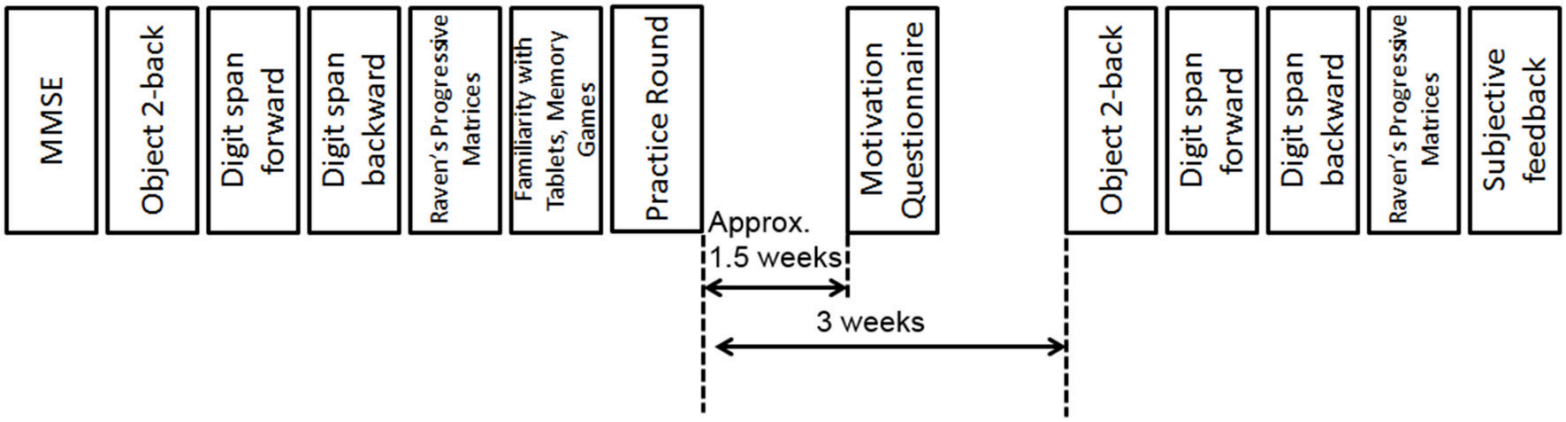
**Study protocol**.

Participants were initially screened by conducting the mini-mental state examination (MMSE). Four standardized working memory and fluid intelligence assessments were administered prior to the study (described in detail in Section Measures). To assess participants' previous experience with memory training games and tablets, they were subsequently asked to fill a questionnaire (Appendix B in Supplementary Material). This was followed by a practice round in which the participants were told about the game and then asked to play a few practice rounds. Once the participants were assured that they could play the game themselves, and if they still agreed to keep the tablet for 3 weeks, they were asked to fill a consent form, and the tablet was handed over, along with a motivation questionnaire that the participants were told to fill out after approximately 1.5 weeks, and a list of Frequently Asked Questions about using the tablet and the game. At the end of 3 weeks, participants were contacted again, and the four standardized assessments were administered again (Figure 1). One of the aims of the study was to gauge participants' compliance in playing a memory training game of their own volition. Therefore, it was explicitly told to the participants that they could play the game whenever and for however long they wished. No frequency or duration of sessions was imposed or suggested. Additionally, participants were not contacted in any way during the 3-week period, although they could contact the test administrator if they had any queries.

### The game

**Figure 2:**
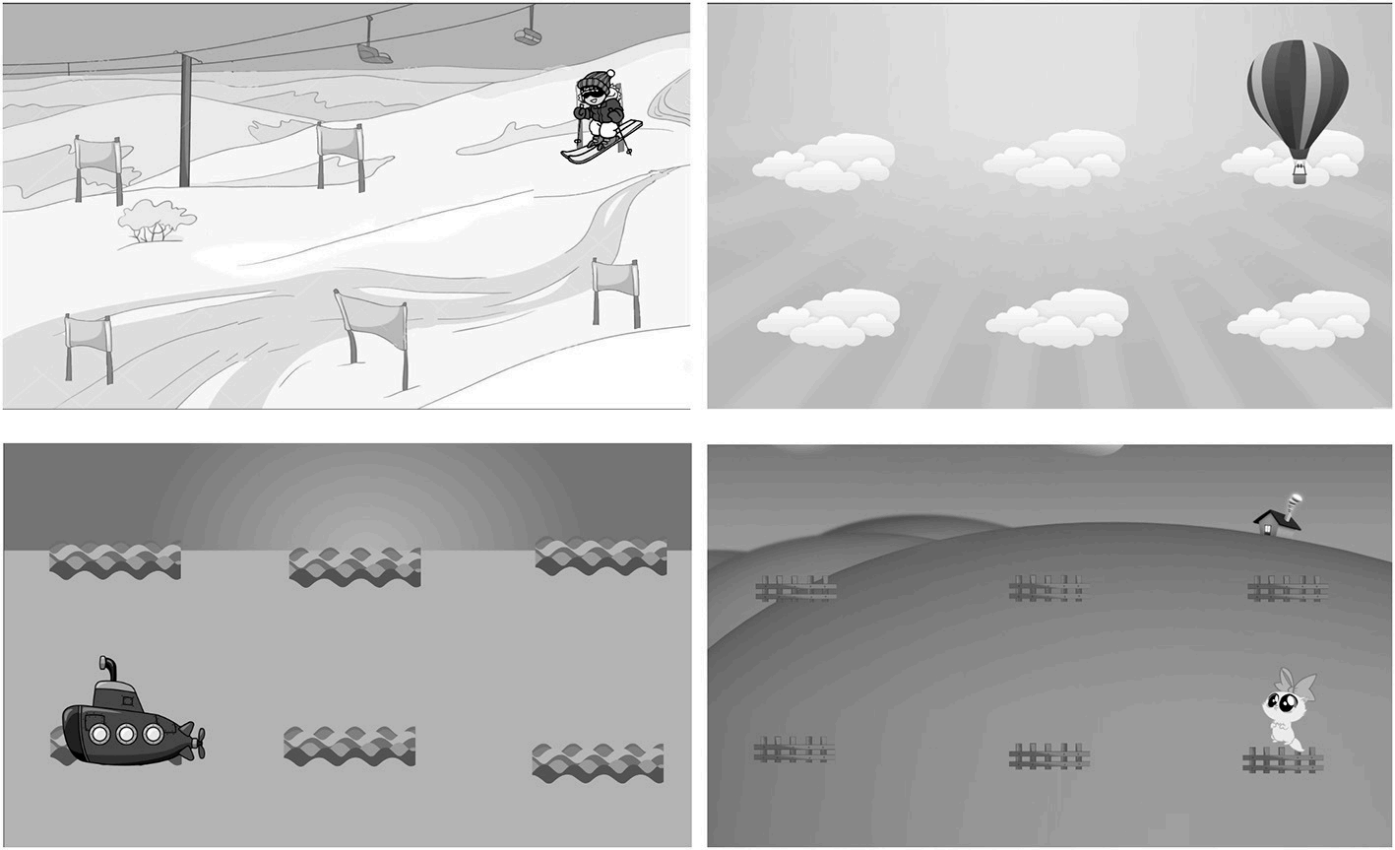
**Screenshots of the four n-back game themes**.

The game used in the present study was a game-like version of the spatial n-back task (Figure 2) (Kirchner, 1958), similar to the one used in Katz et al. (2014). The n-back task has been extensively used in various forms both as an assessment (Kane et al., 2007) and training (Jaeggi et al., 2008) tool for working memory. The spatial n-back game used in the present study presented participants with stimuli at one of six locations on the tablet screen, with a presentation duration of 2 s, and an inter-stimulus interval of 2.5 s. Participants were required to press a “Yes” button if the current stimulus matched the location of the one presented *n* items previously, and a “No” button if the current stimulus did not match. To make the game appealing, four themes were developed in which the actual task was framed, with the stimulus and the location appearing differently depending on the theme: kitten on a fence, balloon in clouds, skier on a slope, and submarine on waves (Figure 2). The game was played in rounds, with each round consisting of 15+*n* trials, and each round consisting of five targets and 10+*n* non-targets, similar to the protocol used in Katz et al. (2014). Two modes of the game were tested, differentiated by distinct implementations of three game elements: difficulty adaptation, rewards, and visual theme.

#### AUTO

Game elements were automatically adjusted in this mode, without user control. Difficulty adaptation was implemented after every round by incrementing *n* by one if there were three or less errors and decrementing *n* by one if there were nine or more errors, with the value of *n* being restricted to the range [1, 4]. The initial value of *n* was set to 2. This was therefore a minimalist version of DDA. Although the difficulty change was done after every round, counting of errors was reset only after a decrementing instance. For example, if the first two rounds had two errors each, *n* would be incremented after both, but with the total number of errors at four, the possibility for *n* to be incremented would not arise again until the number of errors equaled or exceeded nine, in which case *n* would be decremented and the error count would be reset. A feedback message was displayed after every round, informing the participants about their in-game performance, with high in-game performance automatically triggering a motivating message. The feedback was designed to be as informative as possible, in order to motivate users to play further (Burgers et al., 2015). The visual theme was preset to the kitten on a fence theme.

#### USER-CONTROL

USER-CONTROL consisted of exactly the same game, with user control incorporated into the three game elements. There was no automatic difficulty adaptation. Instead, after every round the game computed the desired change in difficulty using exactly the same DDA algorithm as in AUTO, and informed the participant about it. Subsequently the participant could choose either to follow the advice of the game, or to ignore the game's advice and set the value of *n* as they wished. As in AUTO, the initial value of *n* was set to 2. In addition to a feedback message after every round, a certain number of rounds triggered a reward, given in the form of a motivating animation, in line with recommendations that rewards for older adults be intuitive and have minimal text (Fua et al., 2013). Rewards were given according the variable-ratio schedule (Nagle et al., 2014b), in which the number of rounds between two reward-giving instances was set to a random number between ⌈6/*n*⌉ and ⌈9/*n*⌉, where *n* was the current n-back level. The number of rounds before the (*i*+*1*)^th^ reward instance was computed at the *i*^th^ reward instance, with the reward-instance interval for the first reward being initialized to a random number between 3 and 5. Rewards were thus unpredictable and hence potentially more enjoyable (Buitenweg et al., 2012). Additionally, taking into account the current n-back level resulted in rewards being given more frequently at a higher difficulty. It was expected that participants would learn this reward-giving schedule and accordingly set a higher *n* level. Unlike in AUTO, participants could choose among one of the four visual themes (Figure 2) at the start of every game session.

The 21 participants who volunteered to take part in the study were pseudo-randomly assigned to one of the two groups in a way that the two groups would have participants of similar age and gender. These two factors could potentially affect the efficacy of the two modes, and therefore pseudo-randomization was done to prevent imbalance between the modes in terms of age and gender, similar to the widely used concept of *stratified randomization* (Kernan et al., 1999). The idea of stratified randomization was implemented with a brute-force approach in the present study, since the number of participants was not large. An algorithm was designed that randomly “shuffled” participants into the two modes, with a new random seed being used for each shuffle. Shuffles that produced acceptably balanced groups were added to a bin, and one shuffle (assignment of participants into groups) was finally randomly chosen. The final composition of the two groups was as follows:

AUTO: 11 participants, average age = 70.2 years, SD = 5.29 years, 5 females, 6 males

USER-CONTROL: 10 participants, average age = 69 years, SD = 7.2 years, 5 females, 5 males.

### Measures

Two types of measures were considered, related to the two aims.

#### Compliance

1. Motivation: This measure consisted of the following six subscales of the Game Motivation Scale: intrinsic motivation, integrated regulation, identified regulation, introjected regulation, external regulation (this is what other literature refers to as extrinsic motivation), and amotivation (Lafrenière et al., 2012). The value of each subscale was computed from a 17-question questionnaire that participants were told to fill out approximately 1.5 weeks into the study (Appendix A in Supplementary Material).
2. Total duration of sessions: This was measured as the total amount of time participants spent in playing the game over the 3 weeks.
3. Frequency of sessions: In order to gauge how often participants played the game after being told that they could play whenever and however long they wished, the number of gameplay sessions consisting of at least two rounds was counted.

#### In-game performance

Since USER-CONTROL allowed participants to set the n-back level themselves, comparing the n-back level across the modes would not be a meaningful comparison. Therefore, an explicit in-game performance measure was defined as:

$$Performance=\frac{1}{N}\sum_{i=1}^{N}\frac{C(i)}{T(i)}M(i)$$

Here, *N* = number of trials in a session, *C(i)* = number of correct answers in trial *i, T(i)* = total number of stimuli in trial *i*, and *M(i)* = n-back level in trial *i*. The in-game performance number thus computed accuracy of answers, multiplied by n-back level to give a higher weight to a higher n-back level.

**Figure 3:**
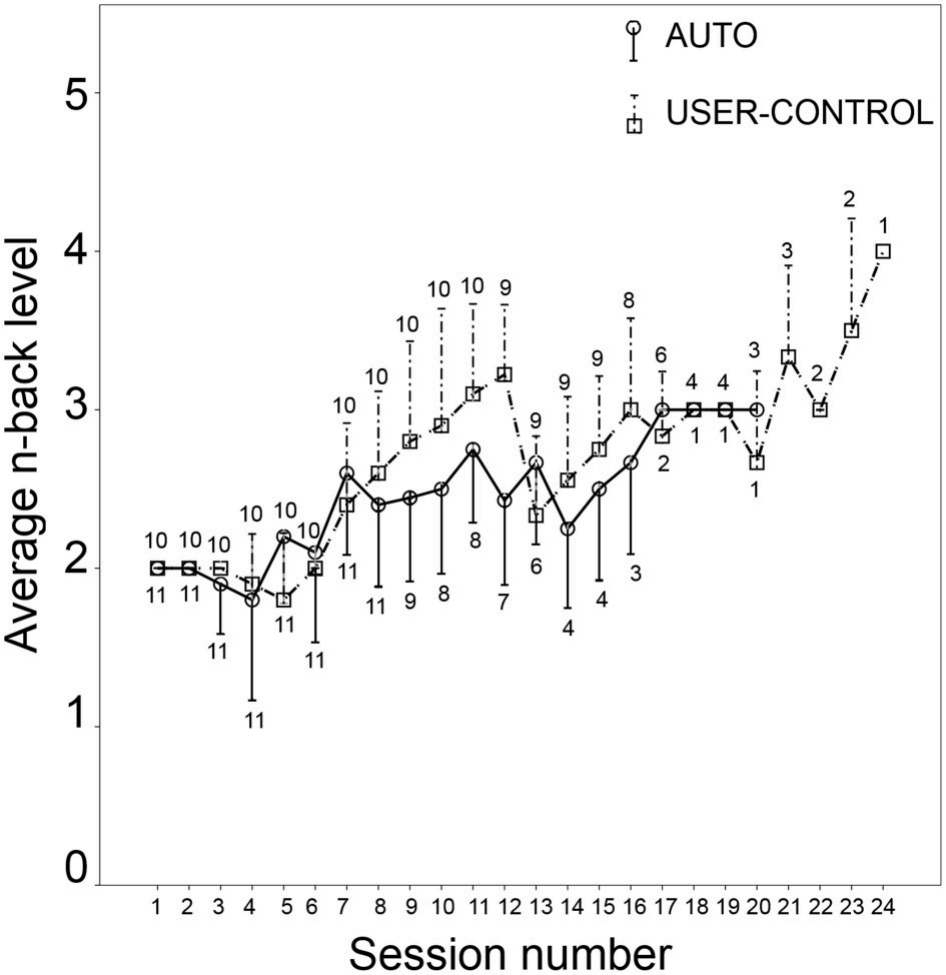
**Average and standard deviation of n-back level in each session for AUTO (circle with solid line) and USER-CONTROL (square with dashed line)**. Number of participants in that session is indicated below the line for AUTO and above the line for USER-CONTROL.

In using the above in-game performance metric, it must be noted that several sessions of low difficulty cannot be fairly compared with a session of high difficulty. In the AUTO mode, n-back level was not under user control. In USER-CONTROL, on the other hand, the user could “cheat” by setting consistently low values of the n-back level, which would presumably translate to better accuracy and consequently higher in-game performance number. To check for this, the average n-back level in each session was compared between AUTO and USER-CONTROL (Figure 3). In the first seven sessions, the n-back level set by users in USER-CONTROL was the same or slightly less than what the game set (Figure 3). However, in all the latter sessions except three, average n-back level set by users was higher than in AUTO. It seems therefore that users refrained from setting excessively low values of *n*, thus validating the suitability of the used in-game performance metric.

#### Transfer effects

In addition to these primary measures, three standardized tests of fluid intelligence and memory span were conducted prior to and after the study to gauge possible near-transfer effects of the two modes.

1. Raven's Advanced Progressive Matrices (RAPM): This is a standard nonverbal intelligence test (Raven and Court, 1998), which is based on perceptual analogies presented in the form of two-dimensional pattern-matching matrices. A matrix of figures was presented in which one position was empty; by deducing the relationship between rows and columns, the participant was required to infer what figure should be in the empty position of the matrix, and select from eight options (Klingberg, 2010). There were 36 patterns; the number of correctly answered patterns in 10 min was used as the outcome measure. Previous studies among the young healthy have found a transfer effect from training on the n-back task to scores on RAPM (Jaeggi et al., 2008), and therefore this measure was included to test if there is a similar transfer effect among healthy older adults.
2. Digit Span Forward and Backward: This is a standardized measure of working memory, often used as part of intelligence tests (Saklofske and Schoenberg, 2011) and to test transfer effects on working memory (Dahlin et al., 2008; Borella et al., 2010). Participants were presented with a sequence of digits of increasing length (three to eight), which they had to recall, first in the same order (Forward), and then in reverse order (Backward). One point was awarded for each correct answer, with the maximum possible score being five in each type.
3. Object 2-back test: This was the transfer test closest to the actual training game. Participants were presented with a sequence of images one at a time, with a presentation time of 1.5 s and an inter-stimulus interval of 2.5 s. Participants were required to press a “Yes” button if the currently displayed image was the same as the one displayed two items previously; otherwise they had to press a “No” button (Katz et al., 2014). There were three rounds of 15 trials each, with the percentage of correct responses being considered.

### Data analysis

Participants' experience with tablets and memory training games (Appendix B in Supplementary Material) were coded by assigning 0 in case of no familiarity at all, 1 if the answer was “less than once a week,” 2 if the answer was “1–2 times a week,” 3 if the answer was “3–4 times a week,” and 4 if the answer was “everyday.” Participants' familiarity with the n-back game was coded as 0 if not familiar, and 1 if familiar. Univariate ANCOVAs were conducted to test the effect of mode on the outcome measures after controlling for age, previous experience with tablets, previous experience with memory training games, and familiarity with the n-back game. The four standardized tests were analyzed by considering the difference in the post- and pre-test scores. Additionally, Pearson product-moment correlations were run on the independent and dependent variables.

In-game performance was also analyzed for each session played by the participants. Of course, since participants were free to play whenever they wanted, the number of sessions played differed. The maximum number of sessions played by a participant in AUTO was 20; in USER-CONTROL it was 24. There was one instance where four participants, two each in AUTO and USER-CONTROL, played twice in 1 day; in all other instances, participants played no more than once every day.

## Results

**Figure 4:**
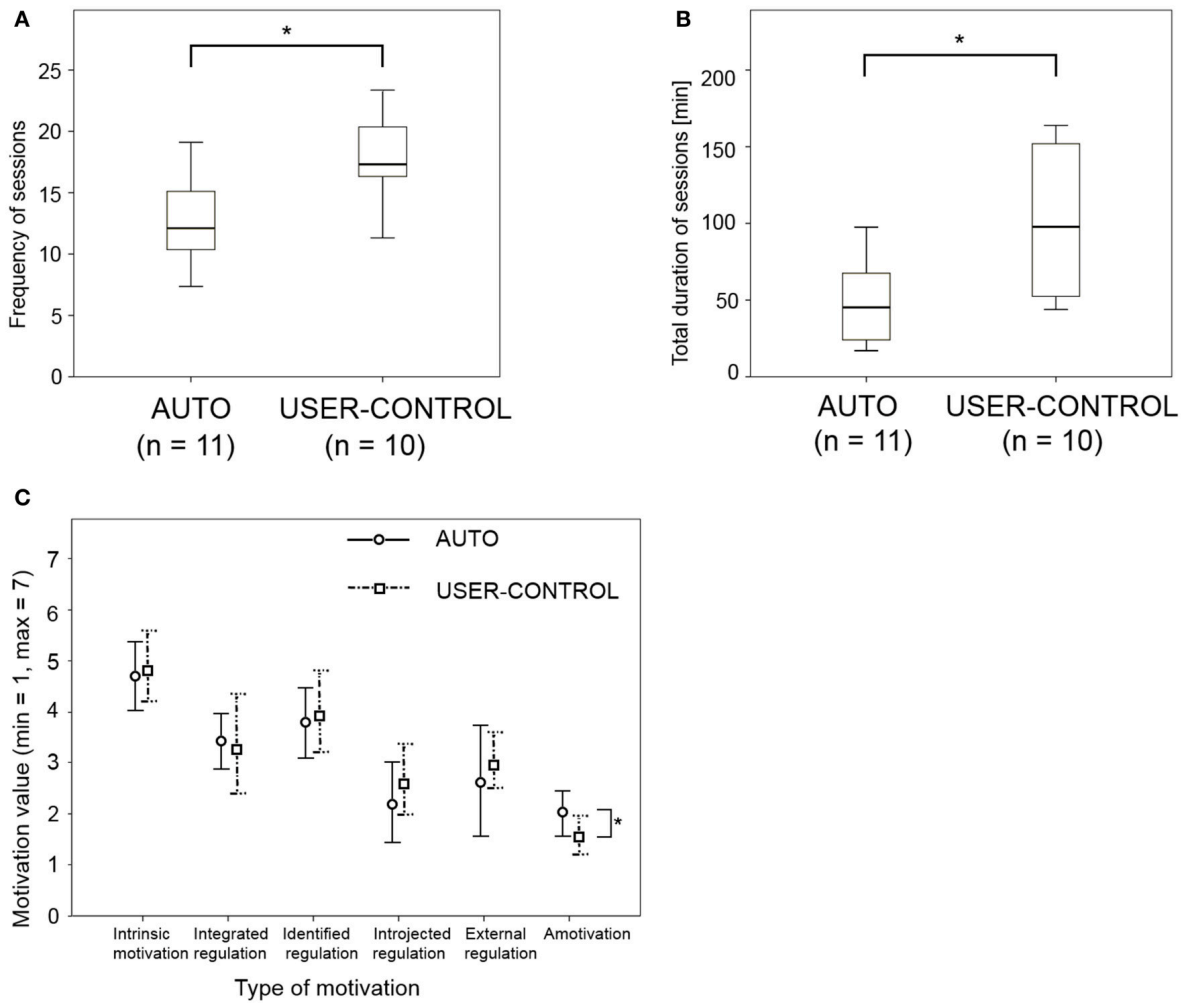
**Results of compliance measures**. **(A)** Box plot of frequency of sessions; **(B)** box plot of total duration of sessions; **(C)** values of the six motivation subscales assessed after 1.5 weeks. Differences marked with a ^*^are significant at the *P* < 0.05 level.

**Figure 5:**
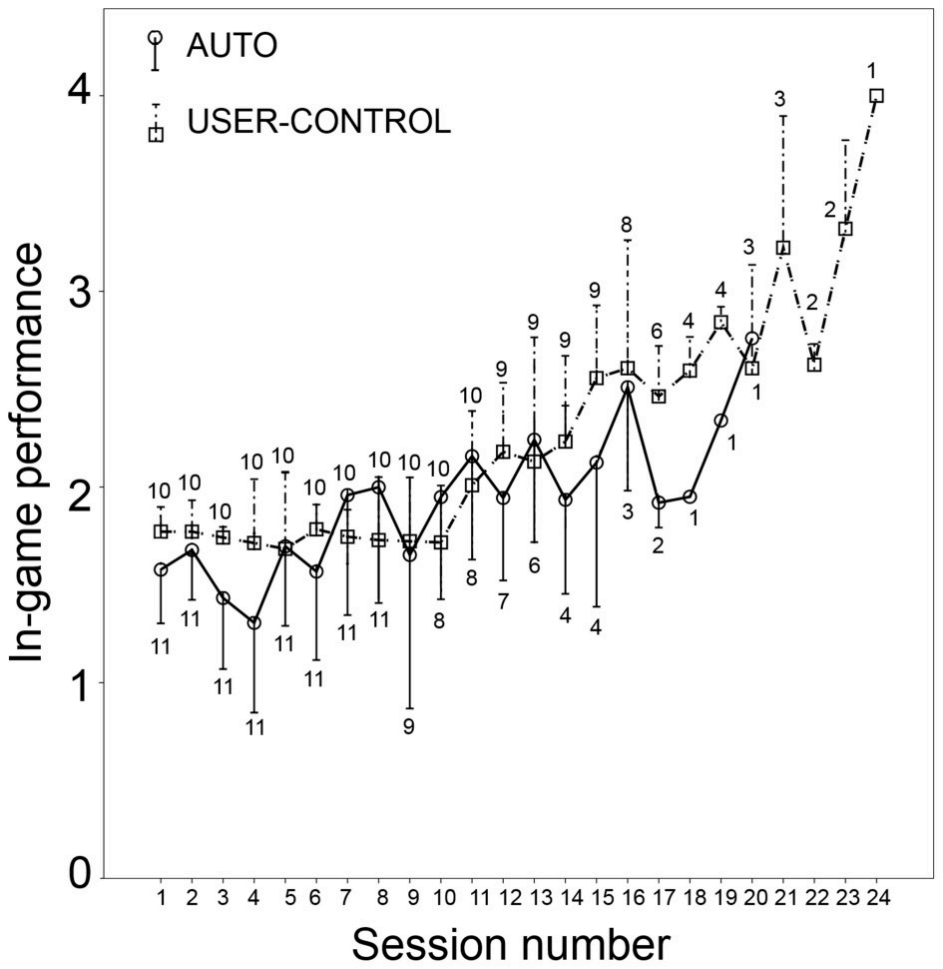
**Average and standard deviation of in-game performance in each session for AUTO (circle with solid line) and USER-CONTROL (square with dashed line)**. Number of participants in that session is indicated below the line for AUTO and above the line for USER-CONTROL.

**Figure 6:**
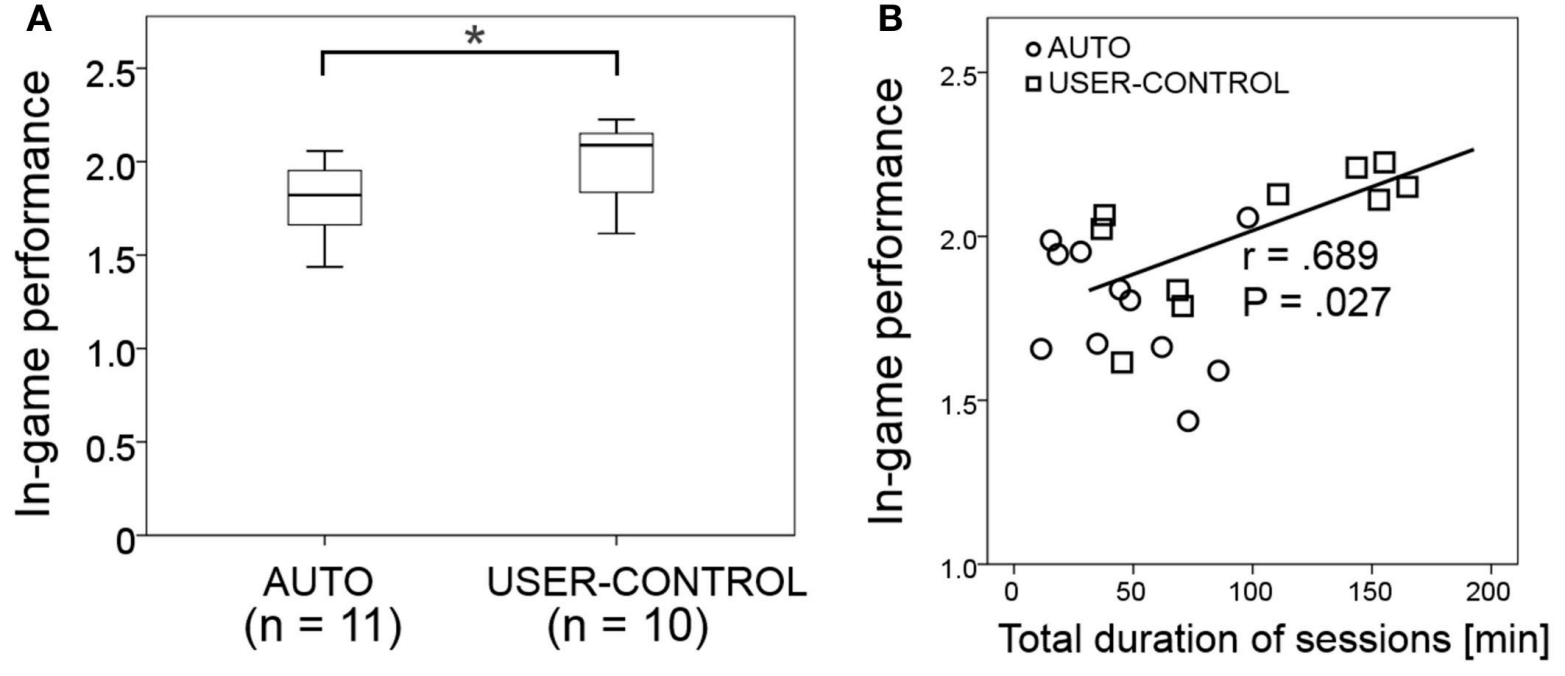
**Results of in-game performance. (A)** Overall average in-game performance in the two modes; difference marked with ^*^are significant at the *P* < 0.05 level. **(B)** Correlation of in-game performance with total duration of sessions, with the correlation coefficient and *P*-value for USER-CONTROL indicated next to its correlation line (no significant correlation existed for AUTO).

**Figure 7:**
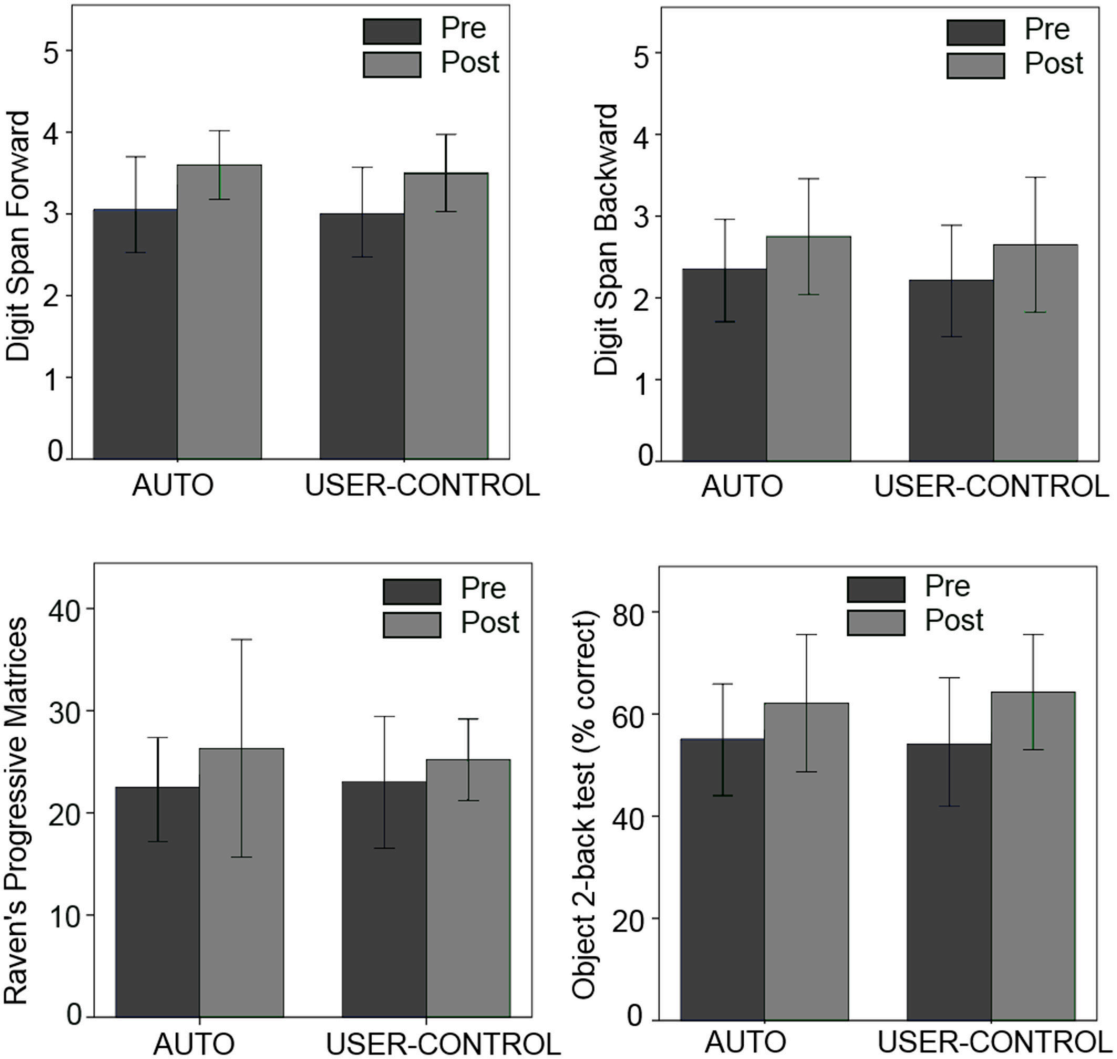
**Pre- and post-study scores in the four standardized tests for the two modes**.

The compliance measures of frequency of sessions [*F*_(1, 15)_ = 6.4, *P* = 0.021] and duration of sessions [*F*_(1, 15)_ = 5.01, *P* = 0.038] were significantly higher in USER-CONTROL than in AUTO, after controlling for age, previous experience with tablets, previous experience with memory training games, and familiarity with the n-back game (Figure 4). Of the six motivation subscales, significant difference between the two modes was observed only in amotivation [*F*_(1, 15)_ = 12.67, *P* = 0.002], where it was significantly higher in USER-CONTROL (Figure 4). Overall in-game performance was significantly higher in USER-CONTROL than in AUTO [*F*_(1, 15)_ = 5.84, *P* = 0.027], with significant differences being observed also in six individual sessions (Table 1; Figures 5, 6). Change in score on the four transfer tests exhibited no significant differences between the two modes (Table 1; Figure 7). A Pearson product-moment correlation revealed no correlation between the independent variables of age, previous experience with tablets, previous experience with memory training games, and familiarity with the n-back task, with *P* > 0.05 for each pair, and *r* never exceeding 0.329. A similar correlation analysis was performed on the frequency of sessions, duration of sessions, in-game performance, and average n-back level in the two modes. Duration and frequency of sessions were positively correlated to each other in AUTO, while duration, frequency of sessions, and in-game performance were positively correlated to each other in USER-CONTROL (Table 2; Figure 6). The only correlation exhibited by average n-back level was to in-game performance in both modes (Table 2).

**Table 1:** **Results of univariate ANCOVAs for the different outcome measures**.

**Type of measure**	**Measure**	***F*-value**	***P*-value**
Compliance	Frequency of sessions	6.4^*^	0.021
	Total duration of sessions	5.01^*^	0.038
	Amotivation	12.67^*^	0.002
In-game performance	In-game performance, overall In-game performance in individual sessions (*m* = number of AUTO participants in that session in, *n* = number of USER-CONTROL participants in that session)	5.84^*^	0.027
	Session 3 (*m* = 11, *n* = 10)	6.88^*^	0.017
	Session 4 (*m* = 11, *n* = 10)	5.25^*^	0.034
	Session 14 (*m* = 4, *n* = 9)	8.74^*^	0.008
	Session 15 (*m* = 4, *n* = 9)	9.74^*^	0.006
	Session 16 (*m* = 3, *n* = 8)	5.78^*^	0.027
	Session 17 (*m* = 2, *n* = 6)	5.17^*^	0.035
Transfer effects	Change in score on RAPM	0.09	0.768
	Change in score on Digit Span Forward	0.34	0.567
	Change in score on Digit Span Backward	0.12	0.756
	Change in score on Object 2-Back	1.29	0.271

*F-values of session-wise in-game performance measures are shown only for the sessions where the differences were significant*.

* *Statistically significant at the P < 0.05 level*.

*Covariates: age, previous experience with tablets, previous experience with memory training games, familiarity with the n-back task*.

**Table 2:** **Results of Pearson product-moment correlation analysis for duration of sessions, frequency of sessions, in-game performance, and average n-back level in the two modes**.

**Mode**		**Frequency of sessions**	**Average n-back level**	**In-game performance**
AUTO	Duration of sessions	*r* = 0.689^**^	*r* = −0.201	*r* = 0.026
		*P* = 0.001	*P* = 0.579	*P* = 0.439
	Frequency of sessions		*r* = 0.123	*r* = 0.019
			*P* = 0.734	*P* = 0.346
	Average n-back level			*r* = 0.757^*^
				*P* = 0.011
USER-CONTROL	Duration of sessions	*r* = 0.802^**^	*r* = 0.458	*r* = 0.689^*^
		*P* = 0.005	*P* = 0.183	*P* = 0.027
	Frequency of sessions		*r* = 0.141	*r* = 0.818^**^
			*P* = 0.502	*P* = 0.004
	Average n-back level			*r* = 0.707^*^
				*P* = 0.022

** *Correlation is significant at the 0.01 level (2-tailed)*.

* *Correlation is significant at the 0.05 level (2-tailed)*.

## Discussion and conclusion

Too often, games for cognitive training are implemented with a focus on the training tasks; game design features are considered secondary (Charsky, 2010). This results in games that are not only dull to play, but also do not produce the desired training effect (Van Eck, 2006). Additionally, studies on cognitive training games frame the training sessions in rigid, regimen-based settings, where, unlike the real world, compliance is not an issue. Previous studies on cognitive training games do not consider specific implementations of game elements, nor do they take into account compliance as a factor (Prins et al., 2011; Hawkins et al., 2013; Katz et al., 2014). The present study is a first step in filling this gap, and the results should lend an insight into the effect of a specific combination of implementations of game elements on compliance and in-game performance in cognitive training games.

### USER-CONTROL vs. AUTO

The present study investigated the combined effect of two specific sets of implementations of three distinct game elements: difficulty adaptation, rewards, and visual themes. In the USER-CONTROL mode, the three elements could be manipulated by users, while in the AUTO mode, the three elements were adjusted automatically by the game. While the design of the present study does not allow speculation about the individual effect of the three elements, the combined effect of giving user control was significant, both in compliance and in-game performance.

#### Compliance

A combination of incorporating user control into difficulty adaptation, visual theme, and rewards, resulted in significantly higher duration of sessions and frequency of sessions as compared to automatically adjusting the three elements, indicating that providing high user control improved compliance. However, motivation exhibited significant difference only in one dimension (amotivation), which is not conclusive. Thus, **Q1** could be answered only partially in the affirmative.

Participants' intrinsic motivation in both modes was high, and was unaffected by mode, indicating that even though participants played less often in AUTO, they still enjoyed the game. Introjected regulation, which is doing something due to anxiety or guilt (Lafrenière et al., 2012) was low in both modes, suggesting that participants did not feel any pressure or obligation to play the game. Amotivation was significantly less in USER-CONTROL, although the difference between the two modes was quite small, indicating that the higher compliance in USER-CONTROL is not simply due to a difference in amotivation. The absence of observed differences in the motivation sub-scales might also be due to the low sensitivity of the 17 questions used to derive the sub-scale values (Appendix A in Supplementary Material). Integrated regulation, which is indicative of how much an activity is aligned with a person's life goals (Lafrenière et al., 2012) was also low, suggesting that participants did not think that the game would help them with their personal goals, which was expected from a healthy, cognitively unimpaired sample. The low value of integrated regulation, and the fact that very few participants in USER-CONTROL, and none in AUTO, played every day, gives weight to the idea that participants' compliance might be partly due to novelty of the game, which likely wore off in the latter half of the study. Integrated regulation comes closest to intrinsic motivation in the motivation continuum (Lafrenière et al., 2012), but is much too centered on the player, and perhaps touches on aspects of affective computing, which is a paradigm of human-computer interaction where the computer can respond to the user's emotions (Picard, 1997). A game that aligns itself with a player's personal goals is more likely to be played regularly, and therefore eliciting high integrated regulation seems to be a desirable characteristic in a cognitive training game. Future research could thus focus on designing affective games (Gilleade et al., 2005) for cognitive training.

#### In-game performance

Overall in-game performance on the trained task, measured after every session in the 3-week duration, was significantly better in USER-CONTROL than AUTO, thereby answering **Q2** also in the affirmative. However, since in-game performance was significantly positively correlated to duration of sessions in USER-CONTROL, high in-game performance might simply be due to the greater amount of time spent by participants in USER-CONTROL. Moreover, the four transfer tests, which are better indicators of general cognitive performance, did not exhibit any differences between the modes. Hence, the high in-game performance in USER-CONTROL must be interpreted with caution.

### Subjective appraisal of individual game elements

#### Difficulty adaptation

Giving users the control of difficulty can be tricky because they cannot always be trusted to train themselves at a high difficulty level (Morrison et al., 1992; Nagle et al., 2014a). In spite of this, participants in USER-CONTROL set values of *n* comparable to AUTO, averaging to nearly a value of three in the latter sessions (Figure 3). This has special significance in the present instance because of the nature of n-back, which is generally considered to be a difficult working memory task, involving multiple processes (Jaeggi et al., 2010). Even among the healthy young, accuracy in the visual six-location version drops from nearly 100% for 1-back to almost 60% for 3-back (Jaeggi et al., 2010), with a big difference in perceived difficulty between the n-back levels (Herff et al., 2014). Participants' difficulty choice in USER-CONTROL can be partially attributed to difficulty being the only variable under user control and hence any variety participants would want in the game could be achieved only by trying out different difficulty levels. However, two participants in USER-CONTROL gave the subjective feedback that they liked the control of setting the n-back level, while another reported that the *n* level in each new session should either resume at the same level as the last session, or be settable before the start of the first round. This feedback adds weight to the idea that user control of difficulty, both before and during a game, can, over the long run, result in improved in-game performance on the training task.

#### Rewards

While early research on rewards concluded them to be detrimental to motivation (Deci et al., 1999), recent studies and meta-analyses have refuted the all-pervasive negative impression of rewards (Eisenberger and Cameron, 1996; Cameron et al., 2001). In the present study, rewards were given according to the variable-ratio reward schedule (Nagle et al., 2014b), with an added element of difficulty-dependent reward anticipation. Participants were expected to internalize the reward-giving behavior, with the anticipation of rewards, rather than the rewards themselves, being the driver of continued gameplay (Lorenz et al., 2015). One participant in USER-CONTROL gave the explicit subjective feedback that they were thrilled every time a reward appeared, and had learnt that rewards appear more frequently at higher values of *n*. Therefore, giving rewards according to a difficulty-dependent variable-ratio schedule could potentially be a factor of increased compliance in training games.

#### Visual themes

Analysis of participants' choice of visual theme in USER-CONTROL indicated that the “skier on hilltop” theme and the “kitten on fence” theme were the most often chosen, with the submarine theme being the least popular. This may reflect the personal preferences of the particular participant sample. Additionally, three participants in USER-CONTROL gave the subjective feedback that they appreciated having a choice of visual theme, indicating that control of manipulating visual elements of a game could increase enjoyment, reinforcing previous findings (Vasconcelos et al., 2012) and suggestions (Katz et al., 2014).

### Transfer tests

While the present work was primarily about game design, four cognitive tests were included to evaluate the effect of mode on possible near-transfer effects: fluid intelligence (Jaeggi et al., 2008) and memory span (Dobbs and Rule, 1989). However, no significant difference was observed between pre- and post-study scores in any of the four tests, nor any significant effect of mode. While means of the post-study scores were higher than the pre-study scores for every test, this might be due to participants' greater familiarity with the test instrument and medium (Goldberg et al., 2015). The lack of significant difference between the post- and pre-study scores in the four tests can be explained by several factors. Studies in which the RAPM exhibited a transfer effect from n-back training used the dual n-back paradigm, which includes an auditory component in addition to the spatial one (Jaeggi et al., 2008). The dual nature of the n-back task has been reasoned to be the driver of the improvement in intelligence tests like RAPM (Jaeggi et al., 2008) and the single n-back task used in the present study might not be sufficient to produce a change in RAPM. Additionally, testing RAPM immediately after training might limit RAPM improvement, since older adults have been found to show a higher RAPM improvement in a delayed posttest as opposed to an immediate posttest after training (Labouvie-Vief and Gonda, 1976). Lastly, the n-back task itself, in any form, might be an insufficient driver of intelligence gain (Colom et al., 2013). A lack of significant improvement on the object 2-back test might be attributed to the short duration between the post- and pretest, similar to Katz et al. (2014). Performance on the n-back task and digit span are not always correlated, with one of the hypothesized reasons being that while the digit span task is classically conducted aurally, the n-back task is visually presented, leading to a mismatch in the mental strategy used by participants (Miller et al., 2009). In the present study, the digit span test was also visually presented, and therefore a lack of improvement in digit span scores, and a lack of correlation of digit span change with n-back performance, might be more due to the inability of digit span to reflect performance on higher level cognitive processes (Turner and Engle, 1989), including the ones involved in n-back (Jaeggi et al., 2010). While the lack of improvement in the tests is not surprising given the relatively unstructured nature of the study, and does not lessen the main result about the superiority of USER-CONTROL in compliance and in-game performance, the four used tests are still valid measures of fluid intelligence and memory. Therefore, future research could focus on designing games that allow users to play at their own pace, while still affording a level of training that has a high impact on fluid intelligence, for example by including tasks specifically geared toward exercising complex cognitive processes (Miyake et al., 2000).

### Study limitations

The present study has a few limitations. The empirical design of the present study confounds the three considered game design elements. Thus, the present study is unable to determine the significance or contribution of each element. However, the three elements were quite distinct and non-overlapping. Therefore, while the specific contribution of the individual elements could not be determined, it could be argued that the effect of the three was additive, especially given the positive subjective feedback from participants about user control of difficulty and getting rewards. Moreover, all three elements would be required together to make a well-rounded game. A previous study about the differential effect of motivational features in n-back training among school children had concluded that motivational features like visual themes must be “chosen judiciously, and may be unnecessary for driving learning on the core task” (Katz et al., 2014). Results of the present study indicate that appropriately implemented game elements can impact compliance and potentially increase training performance. That being said, future research should focus on investigating the effects of each game element individually, so as to better constrain the design of cognitive training games.

The small sample size and voluntary participation of the 21 subjects, all of them in fairly good health, able to attend and understand lectures (where most of the recruitment was done), and apparently enthusiastic to participate in a 3-week study, limits the generalizability of some of the results, especially about motivation. However, only one of the 21 participants was actually familiar with the n-back paradigm, and therefore the differences between the two modes are assumed to be still valid.

While the rationale behind the home-based and play-whenever-you-wish study design was to investigate willingness to play a fairly difficult and uninteresting game at home, it must be admitted that the study was not wholly free from the Hawthorne effect, which is the problem in experiments that subjects' knowledge that they are in an experiment modifies their behavior from what it would have been without the knowledge (Adair, 1984). None of the participants were contacted in the 3-week duration, to simulate as best as possible an experiment-free environment. However, two participants gave a feedback that they expected to be called up and asked about how the gameplay is progressing, which might indicate that they still felt that they should be under observation. An avenue for future research might be to better simulate a completely experiment-free environment, while still measuring useful data. Additionally, the results of testing a game for 3 weeks, even if in a home-based setting, might not generalize to longer gameplay durations. This is important because previous studies have shown that it is necessary to continue practicing memory training games time to time for extended durations (Ballesteros et al., 2015). Therefore, another avenue for future research could be to investigate ways of seamlessly integrating game play into the lives of older adults for longer durations, for example by using augmented reality (Boletsis and Mccallum, 2014), or more complex tasks (Chein and Morrison, 2010).

Lastly, the present work was not a true randomized controlled trial, since there was no control group. An avenue for future research thus could be to apply a similar study design to a randomized controlled trial.

### Implications of user control in training

Psychologists have long associated user control and choice with increased motivation (Ryan et al., 2006) and enjoyment (Csikszentmihalyi, 1990). Giving users the control to customize different aspects of any media, including games, can provide them with a sense of *agency* or *power*, whereby they feel more invested in the medium and rate the quality of the content highly (Sundar and Marathe, 2010). In games, such user control is an important factor of why players want to play a game repeatedly (Choi and Kim, 2004) and for long durations (Febretti and Garzotto, 2009). In games for learning and training also, user control appears as a factor of making the games more motivating (Baldwin et al., 1991; Cordova and Lepper, 1996) and effective (Wishart, 1990; Cordova and Lepper, 1996). In other areas like motor learning, giving users the control of task difficulty (Andrieux et al., 2012), task duration (Lessa and Chiviacowsky, 2015), or choice of task order (Wulf and Adams, 2014) increases task effectiveness. In fact, studies have shown that even providing relatively meaningless choices, whereby users of a task are told that they can choose one among several options, with the choice not really making any difference to the operation of the task, increases task effectiveness (Lewthwaite et al., 2015). Results of the present study adds to this growing body of research on user control, suggesting that providing such control can increase compliance in cognitive training games.

## Conclusion

Within the spectrum of user control of game elements, giving very high user control of difficulty adaptation, rewards, and visual themes resulted in significantly greater compliance and in-game performance as compared to providing very low control, although transfer tests did not exhibit any differences. While the specific contribution of each individual element cannot be pinned down, subjective feedback from participants about liking user control in difficulty adaptation and anticipation of the difficulty-dependent rewards lends support to the idea that at least for these two elements, user control worked separately. Of the four possible visual themes, two were most frequently chosen, perhaps in alignment with participants' individual preferences. While it is unclear whether choosing a theme of their liking specifically affected participants' compliance or performance, conscious choice of one theme over others indicates desirability and directed use of the given choice.

Results of the present study have several implications for developers of cognitive training games for older adults, and game designers generally. Appropriately designed user control could be extremely effective in increasing compliance in games. User control of difficulty could potentially lead to increased in-game performance on the trained task. Difficulty-dependent reward schedules can heighten user anticipation and could lead to prolonged gameplay. Tablet-based games that older adults can play at home are an effective medium of cognitive training. While two extrema of user control were investigated in the present study, the results could foster research into exploring viable alternatives in the middle of the two ends. Although investigating the effect of user control in improving performance on standardized cognitive tests requires long-term studies, incorporating such control in game elements has the potential to make training games enjoyable and effective.

## Author contributions

AN participated in the study design and participant recruitment, developed the n-back game, supervised the pre- and post-study measurements, and carried out most of the data analysis. RR participated in the study design and data analysis. PW participated in the study design, data analysis, and participant recruitment. All authors jointly drafted and approved the final manuscript.

### Conflict of interest statement

The authors declare that the research was conducted in the absence of any commercial or financial relationships that could be construed as a potential conflict of interest.
