# Supplementary material for: High User Control in Game Design Elements Increases Compliance and In-game Performance in a Memory Training Game
Source: Front Psychol. 2015 Nov 20;6:1774. doi: 10.3389/fpsyg.2015.01774 (PMC4653717; doi:10.3389/fpsyg.2015.01774)
Supplement: Supplementary file 1 [file DataSheet1.PDF]

Appendix A. Game Motivation Scale

Adapted from (Lafrenière et al., 2012).

Participants were asked the following question: ‘‘Why did you play this game?”

Each item was rated on a 7-point Likert scale ranging from 1 (‘‘do not agree at all’’) to 7 (‘‘very strongly agree’’).

Intrinsic motivation

1. Because it is stimulating to play
2. For the pleasure of trying/experiencing new game options
3. For the feeling of efficacy I experience when I play

Integrated regulation

1. Because it is an extension of me
2. Because it is an integral part of my life
3. Because it is aligned with my personal values

Identified regulation

1. Because it is a good way to develop important aspects of myself
2. Because it is a good way to develop intellectual abilities that are useful to me
3. Because it has personal significance to me

Introjected regulation

1. Because I feel that I must play regularly
2. Because I must play to feel good about myself
3. Because otherwise I would feel bad about myself

External regulation

1. To gain in-game awards and trophies or character/avatar’s levels and experiences points
2. For the prestige of being a good player

Amotivation

1. It is not clear anymore; I sometimes ask myself if it was good for me
2. I used to have good reasons, but now I am asking myself if I should continue

3. Honestly, I don’t know; I have the impression that I’m wasting my time

Appendix B. Familiarity with tablets and memory training games

1. Do you regularly use a tablet?

Yes

No

2. If the answer to question (1) is yes, how often do you use a tablet?

Everyday

3-4 times a week

1-2 times a week

less than once a week

3. Do you regularly use a computer?

Yes

No

4. If the answer to question (3) is yes, how often do you use a computer?

Everyday

3-4 times a week

1-2 times a week

less than once a week

5. If the answer to questions (1) or (3) is yes, what do you primarily use a tablet or computer for?

Check email

Read news

View photos

Chat with family members

Other (specify): ______

6. Do you regularly play memory training games of any kind?

Yes

No

7. If the answer to question (6) is yes, how often do you play memory training games?

Everyday

3-4 times a week

1-2 times a week

less than once a week

8. If the answer to question (6) is yes, do you play memory training games using:

Computer

Tablet

Pen-and-paper

Some other means (specify): ________

9. If the answer to question (6) is yes, what kind of memory training games do you play? (please specify)

____

10. Are you familiar with the n-back task?

Yes

No

11. Have you trained on the n-back task before?

Yes

No

12. If the answer to question (11) is yes, when have you trained on the n-back task? (please specify year)

______
